# Monthly Summary

**Published:** 1898-08

**Authors:** 


### Monthly Summary.

THE CARBORUNDUM INDUSTRY.—In the April number of the *Cosmopolitan* for 1894, mention was made of the new abrasive *carborundum*, which was then beginning to attract considerable attention. Since that time great improvement and development have taken place in the process employed in the manufacture of this substance, as is shown by the following facts from a lecture on the works of the Carborundum Company, by Mr. F. Fitzgerald. This company has established an extensive plant at Niagara Falls and derives its electric energy from the Niagara Power Company, to the extent of one thousand horsepower. The crude materials employed in the production of carborundum are sand, coke, sawdust and salt, the first two are the essentials and the last two are auxiliary to more successful working.

The electric furnaces which were originally less than one foot long and one-half a foot high and wide, have grown until their dimensious are sixteen feet long by five feet square. A single charge of such a furnace will yield four thousand pounds of crystalized carborundum. The present furnace is a simple brick box of the interior dimensions just given and is left open at the top. When charged the furnace is filled with a mixture of powdered coke, sawdust and salt, except along the central axis-line, which is occupied by a cylinder of coke. This cylinder is twenty-one inches in diameter and is composed of coarse fragments of coke. It is built up in the furnace during the operation of charging the finer material. This central core acts as a poor conductor for the heavy current sent through the furnace and by its resistance develops the heat necessary to the production of carborundum.

An electric current of enormous volume, beginning with one thousand amperes and reaching as high as four thousand is sent through the coke cylinder for twenty-four hours, at the end of which time the operation is complete. Upon opening the furnace the charge around the core, for the distance of about a foot, is found to be converted into carborundum.

After removal from the furnace, the crystals are broken apart by crushers and subjected for several days to the action of dilute sulphuric acid. The material is then separated, according to size of crystals, into twenty different grades. The dust gives *Flour* carborundum.

The principal use of carborundum is as an abrasive and for this purpose it is usully made into wheels. The cement generally employed to hold the abrasive in the desired form is a kind of porcelain made by mixing the abrasive with pure clay and ground feldspar and firing it for several days in a furnace similar to a porcelain furnace. The cost of carborundum is greater than that of emery but its superior wearing power makes it a cheaper abrasive and an excellent substitute for emery for many purposes. Carborundum is a compound of silicon and carbon; it is insoluble, nearly as hard as the diamond and its use as a good and rapid abrasive promises to be very extended.

ALCOHOL AS A DISINFECTANT.—Recent researches seem to show that absolute alcohol is devoid of all disinfectant properties, whereas proof spirit (50 per cent) gives more tangible results in this direction than either stronger or weaker solutions. Antiseptic substances which, in aqueous solution, are more or less active germicides, entirely lose this property when dissolved in strong alcohol, but, on the other hand, corrosive sublimate, carbolic acid, lysol, and thymol, dissolved in a 50 per cent. solution of alcohol, disinfect better than aqueous solutions of the same strength.—*Med. Press and Circular.*

---

EDUCATION AND STARVATION.—President Harper, of Chicago University, recently, in a public address, stated that scores of students of both sexes in that institution are compelled, in order to pay their way; to struggle along on the scantiest food, and that occasionally they succumb in the effort. He argues strongly the necessity of proper nourishment for the body, if the owner of it is to be of any use in the world. Education will do little for the student if it is obtained at the expense of physical health and strength.

Chicago University is not the only place where physiques are ruined in the mad race for what is called education. All universities can furnish examples of what Dr. Harper calls strong-minded but weak-bodied men, and the schools are doing even more harm in this way than the universities. If the latter are most to blame it is because they set both the example and the pace. To make matters worse they persist in keeping up a survival of barbarism in the form of competition for prizes or relative standing, as if it were possible by any means known to human beings to tell in every case which of two men is the best.

Any reform movement looking to the substitution of more rational ideals of culture, must emanate from the universities, if it is to make rapid headway or some progress. All who teach lower institutions are trained either in a university or by those who have been so trained, and in this way vicious methods are disseminated and perpetuated. But who is to reform the teaching in the universities ?—*Toronto Star.*

HYDRONAPHTHOL IN THE TREATMENT OF DENTAL PULPS.—Dr. Sidney S. Stowell read a paper before the First District Dental Society, State of New York, on the usefulness of hydronaphthol in the treatment of dental pulps dead or alive. Hydronaphthol possesses one-fifth the strength of bichloride of mercury, twice the strength of beta-naphthol and iodoform, three times the strength of salicylic acid and fourteen times the strength of carbolic acid, and is absolutely non-poisonous, non-corrosive and harmless. Dr. Stowell has used this drug constantly for five years to the exclusion of nearly every other antiseptic, and with the utmost satisfaction. When opening up a tooth in which the pulp has been dead for some time, if a twenty-five per cent. alcoholic solution be used none of the dreaded evil results will follow. The penetrating property of the alcohol and its affinity for moisture will carry it ladened with its hydronaphthol in solution to the remotest nook of the pulp chamber and canals, even to the apex and through to the soft tissues, as well as into the tubuli of the tooth. Powdered hydronaphthol, one part mixed with three parts zinc oxid powder, then mixed with the phosphoric acid in the ordinary way, makes the very best capping for exposed or nearly exposed pulps, or as an antiseptic non-irritating lining for all classes of fillings. He first bathes the cavity with the alcoholic solution, and the evaporation of the alcohol leaves a deposit of hydronaphthol on the cavity. Combined with zinc oxychlorid in the same proportions as with the oxyphosphate, it makes a splendid root canal filling. —*Cosmos.*

---

PRESCRIPTION WRITING.—L. D. S.—The "New York Medical Journal" quotes from an article in a druggist's periodical, on the subject of drug names and prescription writing, which may apply in part to dental practice. The prescription pad is not used as much as it should be by the dentist, and in the matter of lotions, etc., for special pathological condition of the oral cavity within the prerogative of our practice, we have many important demands upon our knowledge of prescribing. While we run no such risks as the general physician, even our limitations demand great care to avoid confusion and error, not to speak of the less formidable mistakes of chemical incompatibility. The "New York Medical Journal" quotes the following formula ;

℞ Hydr. chlor., - - gr. xx

Tinct. hyoscy, - - ʒ i.

Aquæ, - - - ʒ ix.

"Which chloride of mercury is intended ?" asks the writer. "The context shows plainly thas neither is wanted but chloral hydrate. Is it not all wrong that human life should thus be trifled with ?"

To which we may add, "Does it not seem all wrong that the laws of a State permit the most dangerous prescriptions to be compounded by young men of meagre and insufficient preliminary and practical training ?" We happen to be familiar with the requirements for the practice of pharmacy in most of the States, and we do not hesitate to affirm that there is not one legalized prescriber, from the Atlantic to the Pacific, in Canada who would be so dense and ignorant as to suppose that a chloride of mercury was meant in the above prescription. The education of the pharmacist, like that of the dentist and the physician in Canada, starts in the right direction by a thorough preliminary examination almost equivalent to that of the Bachelor of Arts of our Universities.—*Dominion Dental Jourual.*

---

A DENTIST in this city made an application of arsenic in the usual manner, and at next sitting attempted to remove pulp, but found it highly sensitive. To hasten matters he applied cocaine with the current and removed the pulp painlessly, but at the next sitting he found the arsenic had been inducted into tissues beyond the tooth. Here was the devil to pay and no funds. Don't say he should have known better—any one might have done the same thing thoughtlessly.—Dr. F. Fletcher, in *Dental Digest.*

